# Feasibility of a telecare solution for patients admitted with COPD exacerbation: screening data from a pulmonary ward in a university hospital

**DOI:** 10.3402/ecrj.v1.24193

**Published:** 2014-06-25

**Authors:** Magnus Gottlieb, Kristoffer Marsaa, Helle Andreassen, Grisja Strømstad, Nina Godtfredsen

**Affiliations:** 1 Thoraxkirurgisk Klinik, Rigshospitalet, Copenhagen, Denmark; 2 Pulmonary Ward, Gentofte Hospital, Gentofte, Denmark; 3 Pulmonary Ward, Bispebjerg Hospital, Copenhagen, Denmark; 4 Department of Respiratory Medicine, Hvidovre Hospital, Copenhagen, Denmark

**Keywords:** COPD, telehealthcare, telecare, telemonitoring

## Abstract

**Background:**

Chronic obstructive pulmonary disease (COPD) is a major cause of morbidity and mortality. Furthermore, the prevalence of COPD is increasing, and it places an increasing burden on health care systems worldwide. Therefore, there is a growing interest in home telecare solutions that can help patients manage their disease at home and thereby possibly reduce the risk of readmission.

**Purpose:**

The primary aim of this study is to assess the feasibility of a telehealth care solution when offered in connection with discharges from a pulmonary ward at a university hospital. Secondary aims are to assess the reasons for the exclusion of patients, and the reasons for patients not consenting to participate, as well as to identify the predictors for consenting or not consenting among the subgroup of eligible patients.

**Methods:**

In this study, all data in the screening log were collected over a period of 10 months.

**Results:**

A total of 462 patients admitted with an acute exacerbation in COPD (AECOPD) were screened. Almost 70% of the patients were excluded, and 49% of the eligible patients did not consent. Thus, only 15.6% of the screened patients were included. No significant differences were found regarding known risk factors of readmission between the eligible patients, who were included, and those who did not consent. The only significant difference was that more patients in the group that consented are being followed up in our outpatient clinic, notably 84% versus 55.7% (p<0.001), suggesting that this telehealthcare solution is 25 more appealing to those patients who are already being followed up in the outpatient clinic.

**Conclusion:**

These findings emphasize the importance of designing telecare solutions that allow for the inclusion of the actual population of patients admitted with AECOPD.

Chronic obstructive pulmonary disease (COPD) is a major cause of morbidity and mortality, and its prevalence is increasing (1, 2). There are approximately 24,000 acute COPD-related hospital admissions every year in Denmark, and the readmission rate is 24% (3). Furthermore, COPD places an increasing burden on health care systems worldwide (4). Fatigue and dyspnoea are part of the reason why COPD patients are often isolated from social life and, in some cases, decline optional health promotion activities, such as pulmonary rehabilitation, at hospitals (5).

There is a growing interest in home telecare solutions that can help COPD patients manage their disease at home and thereby possibly reduce the risk of readmission (6). The expectation of telemedicine improving and streamlining the treatment of COPD is high, even to the extent that some believe that research in the field is primarily executed by those who are enthusiastic about the potential of telehealthcare (6). The effect of telecare solutions remains controversial. In a Cochrane review, the authors concluded that only a small evidence base has been uncovered to support the use of telehealthcare in COPD (7). Another review concludes that there is a positive effect on the risk of readmission (8). The different interventions that have been tested are very heterogeneous. Interventions range from simple telephone/video consultations to daily telemonitoring of physiological parameters or symptom scores. A well-designed, randomized, multicentre study from the United Kingdom found no effect of telemonitoring in postponing admissions or improvement in quality of life (9). The recruitment of patients differs between studies. While some studies recruit patients from general practice or from outpatient pulmonary clinics, others recruit admitted patients on discharge (9–13).

To our knowledge, no study has previously reported results regarding the feasibility of a telecare intervention, when applied in connection with a hospital admission for acute COPD exacerbation (AECOPD).

Therefore, the aim of this study is to assess the feasibility of an implemented telehealthcare solution, when offered routinely in a pulmonary ward at a university hospital with approximately 850 hospital admissions for AECOPD per year. Secondary aims are to assess the reasons for the exclusion of patients, the reasons for the patients not consenting to participate, as well as to identify the predictors for consenting or not consenting among the subgroup of eligible patients.

## Method

### TELEKOL

TELEKOL is a joint venture project between the pulmonary ward on Bispebjerg University Hospital in Copenhagen, and a call centre at the local healthcare centre (FCOE). FCOE is part of the Health Department of the Municipality of Copenhagen and is responsible for the rehabilitation of patients with chronic illnesses in cross-sector co-operation with general practitioners and hospitals. The call centre is staffed by community nurses with special training in COPD management.

After a pilot project comprising five patients, the project started including patients on April 16, 2012, and it is still active. All patients admitted with AECOPD are screened for possible inclusion in the TELEKOL project, before being discharged. Patients who are found eligible are invited to participate in the TELEKOL project. For those who are not included, the reason for exclusion or for not consenting to participate is recorded. The criteria for inclusion are that the patient must be ≥65 years of age, diagnosed with COPD, and have an AECOPD or acute respiratory failure or pneumonia. The age criterion is due to the fact that 65 years is the age limit for receiving community care in Denmark. The diagnosis of COPD is based on spirometry: FEV1 <80% of the expected value and FEV1/FVC < 0.70. If no spirometry is available, a patient can still be included, if a pulmonary specialist assesses that the patient is likely to have COPD based on his/her symptoms and medical history. If the patient consents, he/she is introduced to the telemonitor and instructed on how to perform pulsoximetry. The day after the discharge of the patient, the telemonitor is installed in the patient's home. The intervention lasts for 14 days (10 working days) and consists of six 30-minute video conference sessions with a nurse from the call centre. During a session, the nurse assesses pulse oximetry (SpO_2_ and heart rate), breathing pattern, level of consciousness, and inhalation techniques. The nurse asks about changes to the patient's cough, sputum, chest pains, and leg oedemas. Finally, the nurse can give advice on exercise, diet, and smoking cessation. If a patient experiences a problem that cannot be solved by the nurse at the call centre, the nurse has the possibility of contacting a pulmonary physician on the pulmonary ward for advice during weekdays (from 8:00 am to 3:00 pm). Alternatively, the nurse can request a home visit performed by a homecare nurse from the homecare unit under the municipality of Copenhagen. If the patient is readmitted, before the intervention period of 14 days has ended, or the patient for some reason is not able to or will not continue, the telemonitor is collected and the intervention is terminated. Eight weeks after the intervention has stopped, the patient can be included again, if admitted with AECOPD. The purpose of the TELEKOL project is to improve the quality of life for the discharged patient with COPD, to support self-care, to improve co-operation between health sectors, and to reduce the risk of readmission for the discharged patients with COPD.

### Collection of data

During the first months after the initiation of TELEKOL, the collection of data was not systematic. For this reason, only data regarding patients screened for inclusion from July 1, 2012, was included in this study. A total of 650 patients were screened for inclusion between July 1, 2012, and June 31, 2013. Many patients were screened more than once, because they were readmitted. After removing all doubles, the database consisted of 462 patients. Reasons for exclusion or for not consenting were collected from the screening log. Many of the excluded and non-participating patients could have been excluded for more than one reason. Only the main reason for not attending was included in the analysis.

Concerning the group of eligible patients, all available information from the individual hospital files was included in the analysis. The principles, by which variables were chosen, were that the information on all or almost all patients had to be accessible in their hospital journals, and that the variable had to be a predictor of readmission, or an indicator of the general function level of the patient or the health-related behaviour. It was also recorded whether or not the patients were followed in Bispebjerg Hospital's pulmonary outpatient clinic before and/or after the admission.

### Statistics

SPSS 21 was used to analyse the data. Comparisons between the groups were done with a Chi-square test for binominal variables and with a t-test to compare the means for continuous variables.

### Ethics and consent

All included patients submitted a written consent form regarding the participation in TELEKOL. Since TELEKOL is not designed as a clinical trial, but as an implementation of a treatment, no ethical approval was needed.

## Results

**Fig. 1:**
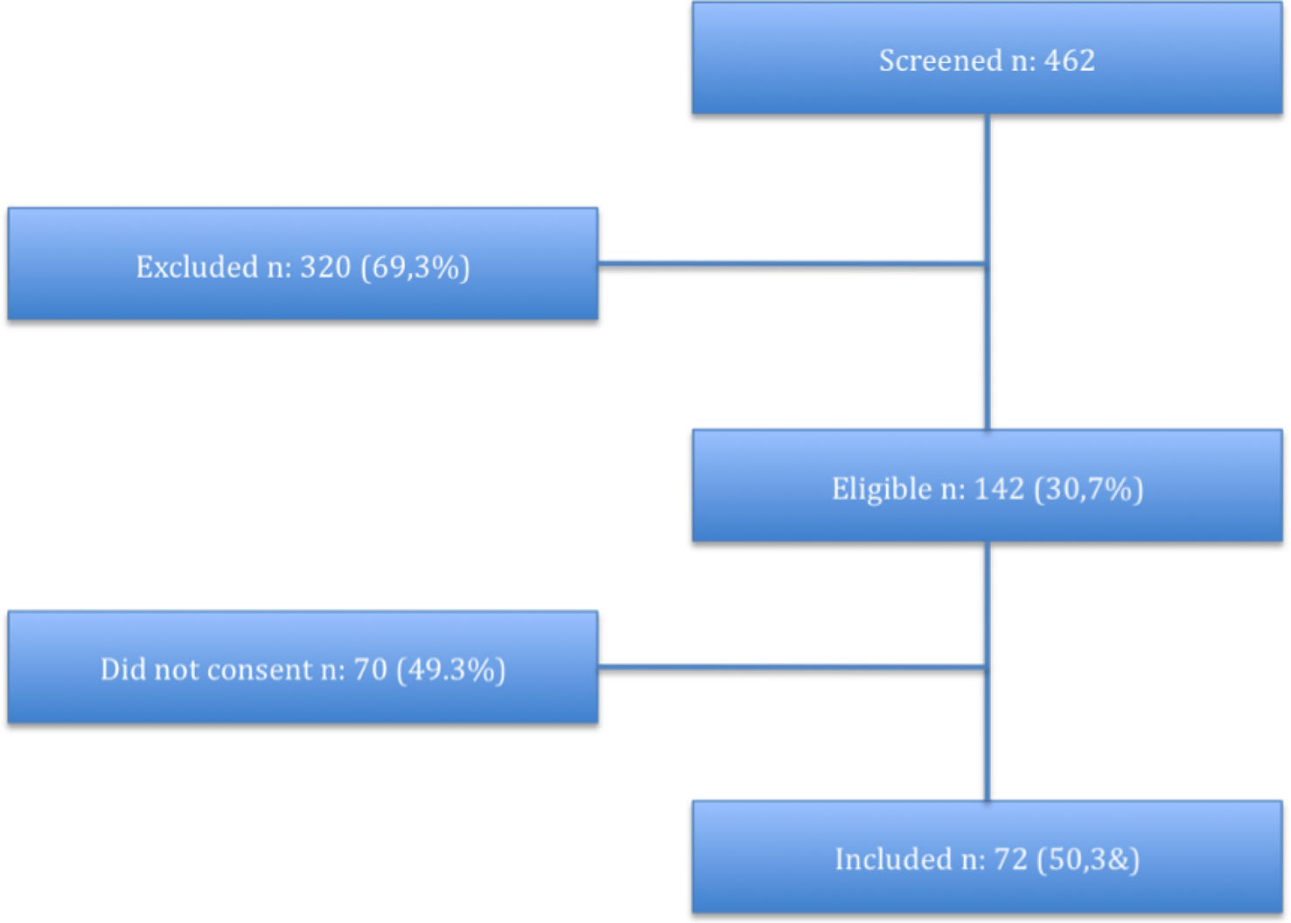
Flowchart of patients that were screened for inclusion in TELEKOL.

Figure 1 shows a flowchart of all 462 patients who were screened for possible inclusion in TELEKOL, of which 142 (30.7%) were found to be eligible. Of these patients, 72 (50.7%) consented. Finally, a total of 15.6% of the screened patients were included.

Table 1 shows the recorded reasons for exclusion. The major reasons for exclusion were that the patients were younger than 65 years (34.4%) and were not to be discharged to their own residence (25.6%).

**Table 1:** Reasons for exclusion

Died before discharge N (%)	27 (8.4%)
Age<65 years old N (%)	110 (34.4%)
Not discharged to own residence N (%)	19 (5.9%)
Not able to cooperate N (%)	36 (11.6%)
Transferred to another hospital department N (%)	27 (8.4%)
COPD was not the main health issue N (%)	18 (8.4%)
Not resident in the municipality of Copenhagen N (%)	82 (25.6%)
Total N (%)	320 (100%)

Table 2 shows the reason given by eligible patients for not consenting to TELEKOL. Around 34.3% are listed as ‘other reasons’. This category mainly consists of patients, who did not give any specific reason not to participate, but also includes reasons that could not be categorised elsewhere. Aside from this, the most frequent reason was ‘I feel too fatigued to participate’ (27.1%). Five patients (7.1%) were eligible, but discharged, before they were introduced to TELEKOL. This can occur if medical wards are very busy, or if the patients are discharged during evenings or weekends.

**Table 2:** Reasons stated by eligible patients for not consenting

Technical barrier	6 (8.6%)
Too fatigued to participate	19 (27.1%)
Found the intervention period to be too short	3 (4.3%)
Other reasons	24 (34.3%)
Eligible, but discharged before they were introduced to TELEKOL	5 (7.1%)
Do not feel any need for TELEKOL	13 (18.6%)
Total	70 (100%)

Table 3 shows the variables that were found in the hospital files for eligible patients. The patients who consented were compared to those who did not. The only significant difference found was that a larger number of patients in the group, who consented, were followed up in our outpatient clinic, notably 84% versus 55.7% (p<0.001).

**Table 3:** Characteristics of eligible patients

Variable	Included	Not included	p
Age, years (SD)	74.4 (6.4)	76.4 (6.4)	0.098
Male, N (%)	26 (44.1)	33 (55.9)	0.182
Spirometry:			
FEV1≥80 predicted N (%)	3 (4.1)	1 (1.42)	0.715
50%≤FEV1<80% predicted N (%)	25 (34.7)	23 (32.9)	
50%>FEV1≥30% predicted N (%)	23 (31.9)	22 (31.4)	
FEV1<30% predicted N (%)	15 (28.8)	14 (20.0)	
No spirometry available N (%)	6 (8.3)	10 (14.3)	
LTOT (long-term oxygen treatment) N (%)	14 (19.4)	17 (24.3)	0.545
Followed in hospital pulmonary outpatient clinic N (%)	61 (84.75)	39 (55.7)	<0.001
Mean length of hospital stay, days	5.72	6.96	0.260
Hospital admissions within 1 year prior to admission, mean	0.86	0.59	0.312
Patients receiving homecare			
None N (%)	35 (48.6%)	35 (50)	0.880
< Once per week N (%)	19 (26.4%)	15 (21.4)	
At least once per week but not daily N (%)	2 (2.8%)	3 (4.3)	
Daily N (%)	16 (22.2)	17 (24.3)	
No. living alone N (%)	54 (75%)	43 (61.4)	0.082
Smoking N (%)	31 (43.1%)	28 (40.0)	0.712

## Discussion

In the study, which TELEKOL is inspired by, 578 admitted patients with AECOPD were screened to get the study population of 102 (14). This also applies to other trials that assess different telehealthcare solutions for patients admitted with exacerbation in COPD after discharge from a hospital (10, 15). Many patients have to be screened, in order for a few patients to be included. Other authors failed to report as to how many patients they screened for inclusion (12, 16). The main finding from this study is that the TELEKOL solution in combination with the chosen exclusion criteria is not feasible. Too few patients were included. When only 15.6% of the patients are included, even a significant effect of the intervention will only have an impact on few patients admitted with AECOPD. Hence, it will be very difficult to show a noticeable reduction in the patient load generated by readmission of COPD patients. Table 1 indicates that the major loss of patients is due to the exclusion criteria. Three of the exclusion criteria have been chosen for bureaucratic reasons, as the patients do not fall under the responsibility of the local healthcare centre FCOE, for example, not discharged to their own residence (25.6%), age <65 years (34.4%), not resident of the municipality of Copenhagen (5.9%). These numbers could be reduced by changing the exclusion criteria. However, it seems likely that some of the patients, who were discharged to nursing homes and temporary rehabilitation homes, would still have been excluded as they were not able to cooperate with TELEKOL due to their physical and/or mental status; 11.6% of the patients were excluded, because it was assessed that they were not able to cooperate. In most cases, this was due to problems with language, weak senses (hearing/sight), dementia, or psychiatric illness. These findings emphasize the importance of choosing exclusion criteria that allows for the majority of patients to participate in telehealthcare solutions, and that trials testing telehealthcare solutions should have exclusion criteria that allow for the inclusion of a population that matches the patients admitted with AECOPD.

One half of the patients, who were found to be eligible, did not want to participate. This emphasizes that the process of recruitment and the design of telehealthcare solutions should be considered carefully in order to make it appealing and available to patients admitted with AECOPD. It weakens our conclusion that 34.3% of the patients did not give any reason for not consenting. However, it is notable that 18.6% stated that they did not feel that they needed the proposed telecare solution, and that another 27.1% felt too fatigued to participate. These findings emphasize the need for studies investigating the demand of and expectations from telehealthcare solutions.

The number of previous admissions with AECOPD is a strong predictor of readmission with AECOPD (17–19), likewise with long-term oxygen treatment (19–21), reduced lung function (18, 22), age (18), and smoking (22). A Danish study in 2009 (23) found home care and dependence on self-care to be predictors of readmission. The amount of home care and whether the patients lived alone were chosen as indicators of the general function and of the social situation of the patients. No significant differences were found regarding known risk factors concerning readmission between the patients who consented and those who did not. This means that it is not possible to point out a specific subgroup of COPD patients, which future telehealthcare solutions should target. The finding that patients that are followed in the outpatient clinic are more likely to accept participation in TELEKOL indicates that TELEKOL is more appealing to the patients who are already able and willing to invest time end energy for their own treatment. This theory is supported by the fact that a large proportion of the eligible patients (27.1%) stated that they felt too fatigued to participate. These might be some of the COPD patients who are in need of efficient follow-up after admission with AECOPD.

This study has several limitations. From the start, TELEKOL was not designed as a clinical trial with strict inclusion criteria and data collection. Spirometry was not part of the screening, and hospital records had to be screened to find records of FEV1 and FEV1/FVC. For 16 (11.2%) of the 142 patients who were found eligible, there was no recorded spirometry, indicating an uncertainty about the COPD diagnosis. Also, there was no systematic recording of symptoms or co-morbidity, which would be relevant.

The strength of this study is that it reflects the clinical reality in a busy pulmonary hospital ward with a large flow of patients admitted with AECOPD. This study contains screening data on a large number of unselected patients with AECOPD. These data could be used to guide the design of future telehealthcare interventions as well as trials testing telehealthcare interventions in order to make them feasible to the actual population of patients admitted with AECOPD.
